# Supplementary material for: Nascent polypeptide-Associated Complex and Signal Recognition Particle have cardiac-specific roles in heart development and remodeling
Source: PLoS Genet. 2022 Oct 14;18(10):e1010448. doi: 10.1371/journal.pgen.1010448 (PMC9604979; doi:10.1371/journal.pgen.1010448)
Supplement: S1 Fig — A, Fluorescently tagged hearts using tdtK were imaged at higher magnification in vivo at white pupae stages which displayed intact hearts in control, Nacα-RNAi, and bicaudal-RNAi expressing hearts, suggesting that the heart histolyzes later, when remodeling during metamorphosis. Arrow heads point to the internal valves that separate the larval aorta from the heart. ^ point to the inflow tracts called ostia. B, Pupal dissections at approximately 24-26hr APF stained with phalloidin shows the presence of the fly aorta and heart in controls (left) and with Nacα KD (right) at two. magnifications. Arrowheads point to the presence of a heart tube. (PDF) [file pgen.1010448.s001.pdf]

Supplemental Figure 1

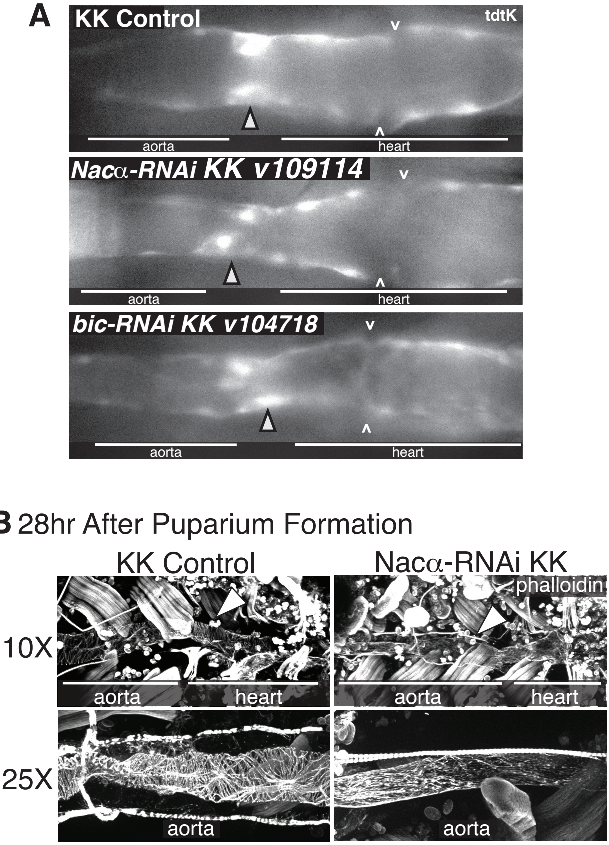

SUPPLEMENTAL FIGURE 1: Cardiac phenotypes following *Nac*α and *bicaudal* knockdown. **A**, Fluorescently tagged hearts using tdtK were imaged at higher magnification in vivo at white pupae stages which displayed intact hearts in control, *Nac*α-RNAi, and *bicaudal*-RNAi expressing hearts, suggesting that the heart histolyzes later, when remodeling during metamorphosis. Arrow heads point to the internal valves that separate the larval aorta from the heart. ^ point to the inflow tracts called ostia. **B**, Pupal dissections at approximately 24-26hr APF stained with phalloidin shows the presence of the fly aorta and heart in controls (left) and with *Nac*α KD (right) at two magnifications. Arrowheads point to the presence of a heart tube.
