# Supplementary material for: Nascent polypeptide-Associated Complex and Signal Recognition Particle have cardiac-specific roles in heart development and remodeling
Source: PLoS Genet. 2022 Oct 14;18(10):e1010448. doi: 10.1371/journal.pgen.1010448 (PMC9604979; doi:10.1371/journal.pgen.1010448)
Supplement: S2 Fig — KD of a second Nacα RNAi line (GD v36017) using Hand4.2-GAL4 driver, led to the presence of an adult heart tube. A, Hearts were dysfunctional, with significantly increased systolic diameter, that led to severely blunted Fractional Shortening. B, Phalloidin staining visualized highly disorganized circumferential fibers (arrowhead). C, KD of a second bicaudal RNAi line (GD v15453) using Hand4.2-GAL4 led to a no heart phenotype. *-indicates missing heart. (PDF) [file pgen.1010448.s002.pdf]

Supplemental Figure 2

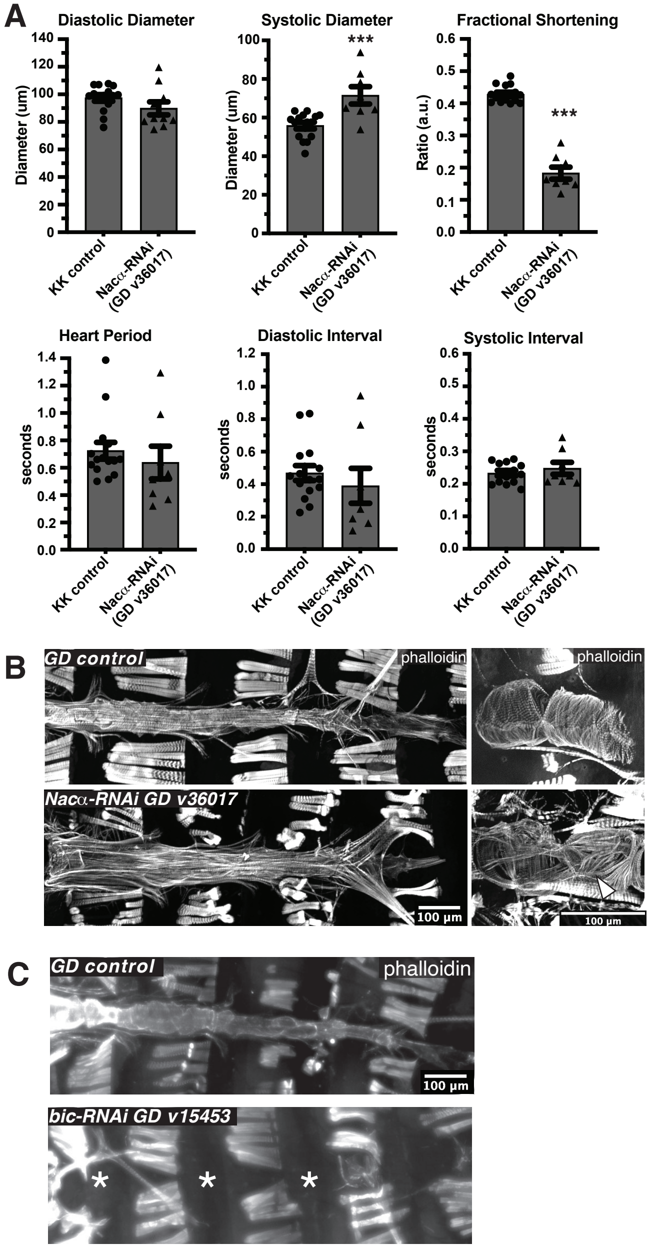

SUPPLEMENTAL FIGURE 2: *Nac*α and *bicaudal* knockdown (KD) using additional RNAi lines. KD of a second *Nac*α RNAi line (GD v36017) using Hand4.2-GAL4 driver, led to the presence of an adult heart tube. **A**, Hearts were dysfunctional, with significantly increased systolic diameter, that led to severely blunted Fractional Shortening.
B, Phalloidin staining visualized highly disorganized circumferential fibers (arrowhead).
C, KD of a second *bicaudal* RNAi line (GD v15453) using Hand4.2-GAL4 led to a no heart phenotype.
*-indicates missing heart.
