# Supplementary material for: Nascent polypeptide-Associated Complex and Signal Recognition Particle have cardiac-specific roles in heart development and remodeling
Source: PLoS Genet. 2022 Oct 14;18(10):e1010448. doi: 10.1371/journal.pgen.1010448 (PMC9604979; doi:10.1371/journal.pgen.1010448)
Supplement: S3 Fig — Posterior structures were more prominent and dilated compared to controls suggesting incomplete histolysis during cardiac remodeling. (PDF) [file pgen.1010448.s003.pdf]

Supplemental Figure 3

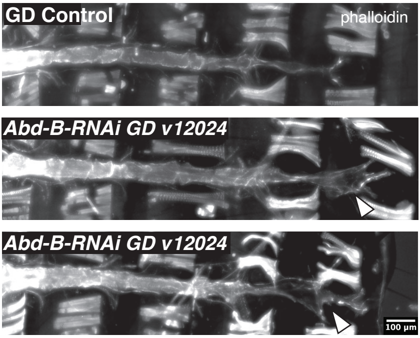

SUPPLEMENTAL FIGURE 3: Knockdown of *Abd-B* using heart specific driver Hand4.2-GAL4 led to intact hearts with posterior ends (indicated by arrowhead). Posterior structures were more prominent and dilated compared to controls suggesting incomplete histolysis during cardiac remodeling.
