# Supplementary material for: Nascent polypeptide-Associated Complex and Signal Recognition Particle have cardiac-specific roles in heart development and remodeling
Source: PLoS Genet. 2022 Oct 14;18(10):e1010448. doi: 10.1371/journal.pgen.1010448 (PMC9604979; doi:10.1371/journal.pgen.1010448)
Supplement: S4 Fig — A, Wing hearts originate from pericardial cells and are required for wing maturation. In controls, proper wing heart function and hemolymph flow leads to adhesion of dorsal and ventral wing layers. B, Normal wing development is also observed with knockdown of Abd-B. C, Knockdown of Nacα using Hand4.2-GAL4 driver leads to fluid filled wing blisters (*) and crumpled wings (^). D, Co-Knockdown of Nacα and Abd-B did not rescue the wing blisters. (PDF) [file pgen.1010448.s004.pdf]

Supplemental Figure 4

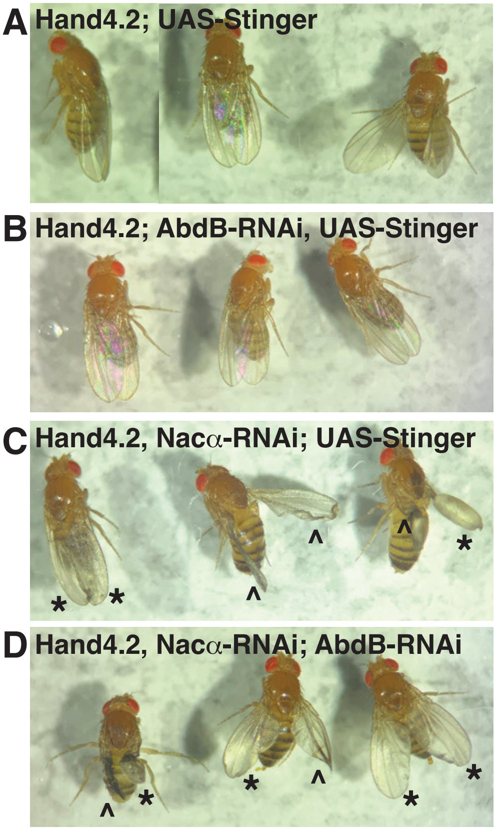

SUPPLEMENTAL FIGURE 4: Wing blister phenotypes. **A**, Wing hearts originate from pericardial cells and are required for wing maturation. In controls, proper wing heart function and hemolymph flow leads to adhesion of dorsal and ventral wing layers. **B**, Normal wing development is also observed with knockdown of *Abd-B*. **C**, Knockdown of *Nacα* using Hand4.2-GAL4 driver leads to fluid filled wing blisters (*) and crumpled wings (^). **D**, Co-Knockdown of *Nacα* and *Abd-B* did not rescue the wing blisters.
