# Supplementary material for: Nascent polypeptide-Associated Complex and Signal Recognition Particle have cardiac-specific roles in heart development and remodeling
Source: PLoS Genet. 2022 Oct 14;18(10):e1010448. doi: 10.1371/journal.pgen.1010448 (PMC9604979; doi:10.1371/journal.pgen.1010448)
Supplement: S5 Fig — A, Still images from video recording of hearts for SOHA analysis. While controls and abd-A KD retained a heart structure, Co-KD of Nacα and abd-A led to a no heart phenotype, indicating an inability for abd-A KD to rescue the loss of the heart caused by Nacα KD. B, Nacα KD led to reduced abd-A levels in the heart. *-indicates absence of heart structure. ^ indicates the presence of ostia structures. (PDF) [file pgen.1010448.s005.pdf]

Supplemental Figure 5

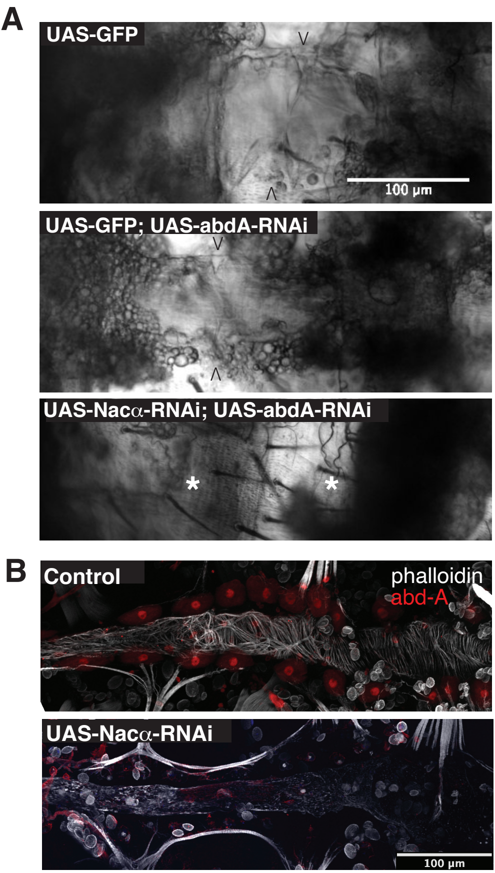

SUPPLEMENTAL FIGURE 5: The Hox gene *abd-A* does not rescue the cardiac phenotype induced by *Nac*α knockdown (KD). **A,** Still images from video recording of hearts for SOHA analysis. While controls and *abd-A* KD retained a heart structure, Co-KD of *Nac*α and *abd-A* led to a no heart phenotype, indicating an inability for *abd-A* KD to rescue the loss of the heart caused by *Nac*α KD. **B**, *Nac*α KD led to reduced *abd-A* levels in the heart. *-indicates absence of heart structure. ^ indicates the presence of ostia structures.
