# Supplementary material for: Nascent polypeptide-Associated Complex and Signal Recognition Particle have cardiac-specific roles in heart development and remodeling
Source: PLoS Genet. 2022 Oct 14;18(10):e1010448. doi: 10.1371/journal.pgen.1010448 (PMC9604979; doi:10.1371/journal.pgen.1010448)
Supplement: S6 Fig — Overexpression of an inhibitor of apoptosis (Diap1). concurrently with Nacα -RNAi does not rescue the loss of the heart. * indicates absence of the adult heart tube. (PDF) [file pgen.1010448.s006.pdf]

Supplemental Figure 6

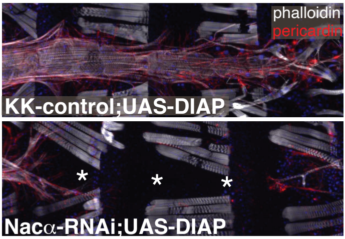

SUPPLEMENTAL FIGURE 6: *Nacα* does not interact with *DIAP1*.
Overexpression of an inhibitor of apoptosis (***Diap1***) concurrently with *Nacα*-RNAi does not rescue the loss of the heart. * indicates absence of the adult heart tube.
