# Supplementary material for: Nascent polypeptide-Associated Complex and Signal Recognition Particle have cardiac-specific roles in heart development and remodeling
Source: PLoS Genet. 2022 Oct 14;18(10):e1010448. doi: 10.1371/journal.pgen.1010448 (PMC9604979; doi:10.1371/journal.pgen.1010448)
Supplement: S7 Fig — Testing interaction of Nacα and Hox gene Abd-B using the cardiac specific tinHE-GAL4 driver by A, functional, B, temporal and C, structural assessment. A, Knockdown (KD) of Nacα (combined with UAS-Stinger::GFP to control for UAS binding sites) using tinHE-GAL4 caused a decrease in both diastolic and systolic diameters that produced a slight but not significant decrease in fractional shortening. KD of Abd-B (combined with UAS- Stinger::GFP) did not produce significant. changes in fractional shortening or diameters compared to control but fractional shortening and diastolic diameters were significantly higher compared to Nacα;Stinger genotype. Combined knockdown of Nacα and Abd-B produced heart parameters that were not different to controls but recapitulated heart function produced by Abd-B KD alone, suggesting that the heart function was rescued. B, Temporal parameters were unchanged with Nacα-RNAi expression. KD of Abd-B lengthened systolic interval compared to controls. Combined Nacα and Abd-B KD displayed longer systolic intervals similar to Abd-B KD alone, suggesting a rescue. C, Phalloidin staining of select genotypes. Compared to controls, Nacα knockdown disrupted circumferential fiber organization creating gaps in the matrix (similar to Fig 2I). KD of Abd-B did not significantly alter circumferential fiber organization. Combined knockdown of Nacα and Abd-B (2 examples shown) improved circumferential fiber organization compared to Nacα knockdown alone. * vs control KKGD. ^ compared to Nacα;Stinger. * p<0.05, ** p<0.01, *** p<0.001. (PDF) [file pgen.1010448.s007.pdf]

Supplemental Figure 7

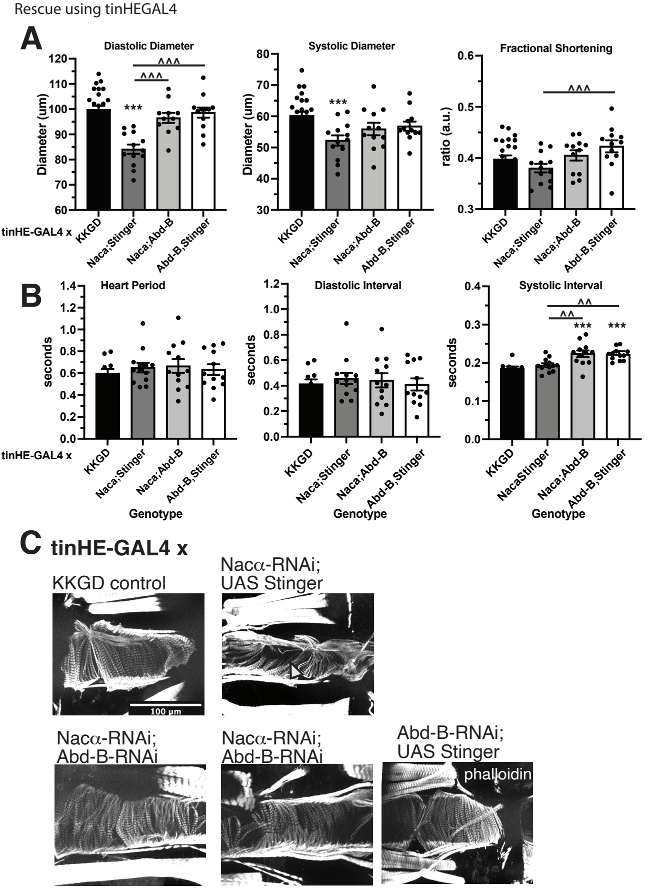

SUPPLEMENTAL FIGURE 7: *Nacα* interactions with *Abd-B* to regulate heart function and structure. Testing interaction of *Nacα* and Hox gene *Abd-B* using the cardiac specific tinHE-GAL4 driver by **A**, functional, **B**, temporal and **C**, structural assessment. **A**, Knockdown (KD) of *Nacα* (combined with UAS-Stinger::GFP to control for UAS binding sites) using *tin*HE-GAL4 caused a decrease in both diastolic and systolic diameters that produced a slight but not significant decrease in fractional shortening. KD of *Abd-B* (combined with UAS- Stinger::GFP) did not produce significant changes in fractional shortening or diameters compared to control but fractional shortening and diastolic diameters were significantly higher compared to *Nacα*;Stinger genotype. Combined knockdown of *Nacα* and *Abd-B* produced heart parameters that were not different to controls but recapitulated heart function produced by Abd-B KD alone, suggesting that the heart function was rescued. **B**, Temporal parameters were unchanged with *Nacα*-RNAi expression. KD of *Abd-B* lengthened systolic interval compared to controls. Combined *Nacα* and *Abd-B* KD displayed longer systolic intervals similar to *Abd-B* KD alone, suggesting a rescue. **C**, Phalloidin staining of select genotypes. Compared to controls, Nacα knockdown disrupted circumferential fiber organization creating gaps in the matrix (similar to Figure 2I). KD of *Abd-B* did not significantly alter circumferential fiber organization. Combined knockdown of *Nacα* and *Abd-B* (2 examples shown) improved circumferential fiber organization compared to Nacα knockdown alone. * vs control KKGD. ^ compared to Nacα;Stinger. * $p<0.05$, ** $p<0.01$, *** $p<0.001$.
