# Supplementary material for: Nascent polypeptide-Associated Complex and Signal Recognition Particle have cardiac-specific roles in heart development and remodeling
Source: PLoS Genet. 2022 Oct 14;18(10):e1010448. doi: 10.1371/journal.pgen.1010448 (PMC9604979; doi:10.1371/journal.pgen.1010448)
Supplement: S8 Fig — Nacα mRNA levels are reduced in adult hearts of HTT flies subject to Nacα KD during pupal stages only. Nacα mRNA levels are also reduced in early pupal hearts of HTT flies subject to Nacα KD during embryonic stages only. (PDF) [file pgen.1010448.s008.pdf]

Supplemental Figure 8

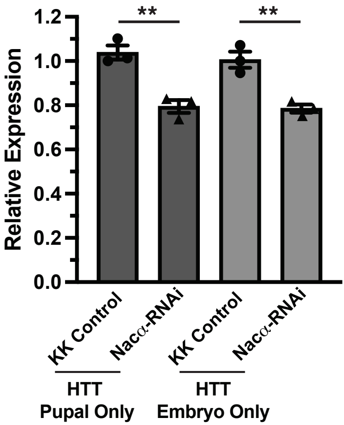

Supplemental Figure 8: **Relative *Nac*α *mRNA* expression measured by Real-Time qPCR.** *Nac*α mRNA levels are reduced in adult hearts of HTT flies subject to *Nac*α KD during pupal stages only. *Nac*α mRNA levels are also reduced in early pupal hearts of HTT flies subject to *Nac*α KD during embryonic stages only.
