# Supplementary material for: Nascent polypeptide-Associated Complex and Signal Recognition Particle have cardiac-specific roles in heart development and remodeling
Source: PLoS Genet. 2022 Oct 14;18(10):e1010448. doi: 10.1371/journal.pgen.1010448 (PMC9604979; doi:10.1371/journal.pgen.1010448)
Supplement: S9 Fig — A, Knockdown (KD) of Nacα, Hox genes or their combination did not produce a significant change in the proportion of endothelial cell (CDH5+). The KD of transcription factors Gata4/6,MyoCD increased the proportion of endothelial cells. B, Representative images of immunohistological staining for select conditions. (PDF) [file pgen.1010448.s009.pdf]

Supplemental Figure 9

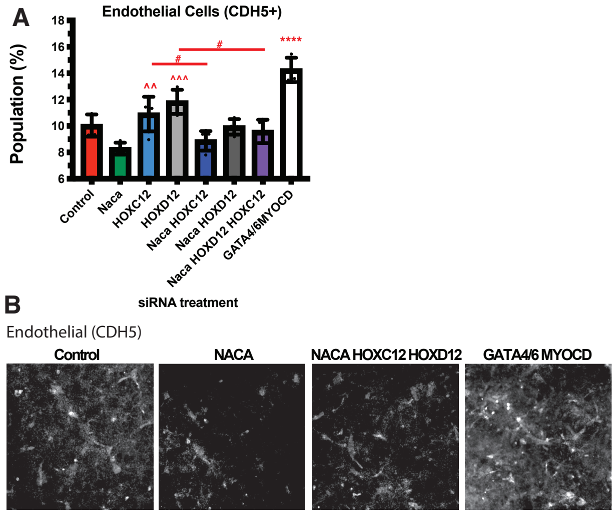

SUPPLEMENTAL FIGURE 9: *Naca*α and *Hox* genes interact to redirect differentiation of Multipotent Cardiac Progenitors (MCPs). A, Knockdown (KD) of *Nac*α, *Hox* genes or their combination did not produce a significant change in the proportion of endothelial cell (CDH5+). The KD of transcription factors *Gata4/6,MyoCD* increased the proportion of endothelial cells. **B**, Representative images of immunohistological staining for select conditions.
