# Supplementary material for: Nascent polypeptide-Associated Complex and Signal Recognition Particle have cardiac-specific roles in heart development and remodeling
Source: PLoS Genet. 2022 Oct 14;18(10):e1010448. doi: 10.1371/journal.pgen.1010448 (PMC9604979; doi:10.1371/journal.pgen.1010448)
Supplement: S10 Fig — Heart tubes were present prior to cardiac remodeling with any of the SRP subunit knocked down using Hand4.2-GAL4, suggesting SRP subunits influence cardiac remodeling into adult structures during metamorphosis. Internal valves separating the larval aorta and heart are marked by arrowheads. (PDF) [file pgen.1010448.s010.pdf]

Supplemental Figure 10

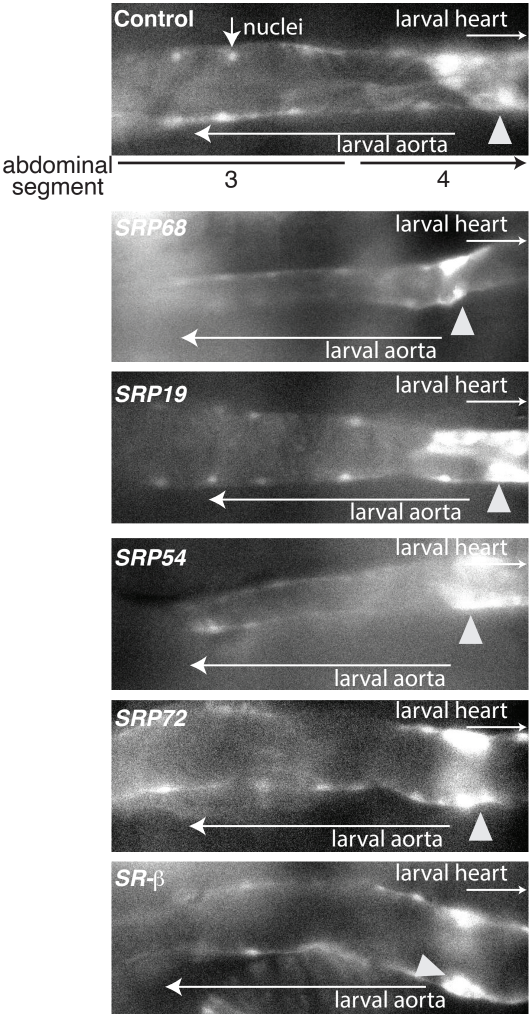

SUPPLEMENTAL FIGURE 10: Images of fluorescently labeled (tdtK) larval aorta and heart (abdominal segments 3 and 4) of White Pupae. Heart tubes were present prior to cardiac remodeling with any of the SRP subunit knocked down using Hand4.2-GAL4, suggesting SRP subunits influence cardiac remodeling into adult structures during metamorphosis. Internal valves separating the larval aorta and heart are marked by arrowheads.
