# Supplementary material for: Nascent polypeptide-Associated Complex and Signal Recognition Particle have cardiac-specific roles in heart development and remodeling
Source: PLoS Genet. 2022 Oct 14;18(10):e1010448. doi: 10.1371/journal.pgen.1010448 (PMC9604979; doi:10.1371/journal.pgen.1010448)
Supplement: S1 Table — * indicates RNAi lines displayed in Figures. BDSC- Bloomington Drosophila Stock Center. VDRC- Vienna. Drosophila Resource Center. (PDF) [file pgen.1010448.s011.pdf]

Supplemental Table 1

Fly gene	Human Gene	DIOPT score	Fly Collection	ID/Stock
Nac-alpha	*NACA*	11	VDRC	KK v109114 *
				GD v36017 *
bicaudal	*BTF3/NACβ*	12	VDRC	KK v104718 *
				GD v15453 *
AbdB	*HOXC12*	3	VDRC	KK v104872
	HOXD12	3		GD v12024 *
	(multiple orthologs)			
UAS-AbdB			BDSC	913
abdA	*HOXC6*	3	VDRC	GD v51900 *
	HOXB6	3	VDRC	KK v106155
UAS-abdA			BDSC	912
SRP9	*SRP9*	13	VDRC	KK v105682 *
				GD v23678
SRP14	*SRP14*	11	VDRC	KK v101444 *
				GD v23422
SRP19	*SRP19*	14	VDRC	KK v106756
SRP68	*SRP68*	14	VDRC	KK v104867 *
				GD v273351
SRP72	*SRP72*	14	VDRC	GD v43978 *
				GD v21641
SRP54	*SRP54*	11	VDRC	GD v51088
SRPRbeta	*SRPRβ*	13	VDRC	GD v5150 *
				KK v110760
UAS-stinger	*nuclear GFP*		BDSC	84277
UAS-Val10-GFP			BDSC	35786
UAS-DIAP	*XIAP*	7	BDSC	6657
tubulin-GAL80ts	*Tubulin*		BDSC	7017
			BDSC	7019
Hand4.2-GAL4	*Hand*			
Hand4.2-GAL4; tdtK				
tinHE-GAL4	*tinman*			
tinCD4-GAL4	*tinman*			
Dot-GAL4	*UDP glucuronosyltransferase family*			

SUPPLEMENTAL TABLE 1: Table of transgenic lines used in the study. * indicates RNAi lines displayed in figures. BDSC- Bloomington Drosophila Stock Center. VDRC- Vienna Drosophila Resource Center.
