# Supplementary material for: Nascent polypeptide-Associated Complex and Signal Recognition Particle have cardiac-specific roles in heart development and remodeling
Source: PLoS Genet. 2022 Oct 14;18(10):e1010448. doi: 10.1371/journal.pgen.1010448 (PMC9604979; doi:10.1371/journal.pgen.1010448)
Supplement: Attachment — Submitted filename: Schroeder_Naca_PLoSGResponsetoReviewersResub.pdf [file pgen.1010448.s015.pdf]

Schroeder, AM et al. Nascent Polypeptide Associated Complex and Signal Recognition Particle have cardiac-specific roles in heart development and remodeling

Point by point responses to the reviewers' comments (in blue font in the manuscript)

Reviewer #1:

1.) In general, the study is well executed. The major scientific concern is that while the authors propose that reduction of Naca/NACA leads to misexpression of Abd-A/HOXC12 in cardiac cells, the molecular mechanism leading to this abnormal regulation remains unclear. The use of a cardiac specific Gal4 driver in fly to KD Naca only in the forming heart provides some support for the link between Naca and Abd-B during development, but there is no data confirming the specificity of this link in other cell types. It is easy to address with another drivers in order to understand if it is a generic or tissue specific function of Naca.

*To address the tissue specificity of Nacα and Abd-B in the heart, we have included observations on the cardiac-specific rescue of the Nacα knockdown defects by Abd-B-RNAi on wing blisters (new **SUPPLEMENTAL FIGURE 4**). In addition to cardiac tissues, Hand4.2-GAL4 also drives expression in the developing wing hearts, wherein Nacα knockdown leads to severe wing blisters, indicating abnormal wing heart function (Togel et al 2008; Lehmacher et al 2009). While combined knockdown of Nacα and Abd-B rescued heart structure, there is no rescue of wing blisters, suggesting spatially restricted activity of this Nacα and Abd-B interaction (to the heart). <u>See lines 253-58.</u>*

*In addition, in **Figure 2** we used a pericardial cell specific driver (Dot-GAL4), which does not express in the cardiomyocytes of the heart tube but only in pericardial cells. We found no overt effects on heart tube structure and function. Therefore, these data provide additional support for a tissue-specific effect of Nacα KD in the heart. <u>See lines 195-203</u>.*

2.) Data with TinCΔ4-Gal4 suggest that expression thresholds of Naca is required in cardiac cells. However, the authors did not address this point by quantifying Naca expression.

While Nacα knockdown levels would play a role in the phenotype, it is not the only factor. Developmental timing of KD should be considered, and differs with each of the drivers used. Therefore, deciding when to measure Nacα levels and whether that timepoint fully dictates the resulting phenotype would be difficult to establish. For example, when we measured Nacα mRNA levels in newly eclosed adults using tinHE-GAL4, which is a weaker driver, we found no significant differences in expression. However, KD with this driver throughout development still produces an adult heart but with quite severe defects (see Fig 2), suggesting that decreased level of Nacα during development may be the reason, but KD is no longer effective with this driver in adult stages (see graph below). We did not include the Nacα expression data with this driver in the revision.

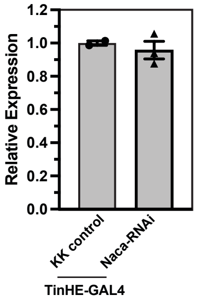

We did, however, include in the revision an experiment where we knocked down Nacα only in pupae and measured Nacα levels in newly eclosed adult hearts (new ***Supplemental Figure 8****). Despite reduced Nacα levels, there is still a rather normal heart present in adults (see Fig. 4D) demonstrating the complicated role of Nacα in the heart and importance of timing in its activity. Conversely, KD only in the embryo produced significant heart loss (Fig. 4F,G), although the KD period was shorter than only during pupal stages. With the embryonic KD regime, we observed reduced Nacα in early pupal hearts (new* ***Supplemental Figure 8****), again demonstrating the long-range effects and likely importance of Nacα in the embryonic hearts. See lines 329-341.*

3.) KD of Naca in the heart led to ectopic expression of Abd-B within the cell. The authors should discuss this point in term of sequence conservation. In the same perspective the authors should discuss the specificity of Naca on Abd-B and not other Hox genes. Does temporal requirement of Naca is only associated with Abd-B misexpression?

We have included data examining the Hox gene abd-A (new ***Supplemental Figure 5****). When Nacα is knocked down, we find by immunostaining, that abd-A levels are reduced in pupal hearts, opposite of the effect on Abd-B. When we concurrently knocked down or overexpressed abd-A along with Nacα knockdown in the hearts, we found no rescue of the heart structures. In fact, overexpression of abd-A led to a worse phenotype of lethality during pupal stages (see lines 258-262). We therefore conclude that there is specificity in interaction between Nacα and Hox genes, particularly with Abd-B in heart morphogenesis. We also mention that the sequence conservation of Hox genes outside of the highly conserved homeodomain very low (see lines 527-529). Using a BlastP sequence alignment tool (blast.ncbi.nlm.nih.gov) there was "no significant similarity" found between Abd-B and abd-A.*

4.) The authors used cardiomyocytes derived from human iPSCs to determine whether similar role of NACA could be observed in other model system. It is clear that NACA siRNA inhibits CM differentiation, but the authors should convince the reader that progenitor cells are not blocked in their initial state. Temporal differentiation of iPSC to CM/fibroblasts should be also determine since premature differentiation of cardiac progenitors has been reported in Hoxb1 mutant mice.

Based on published data characterizing the differentiation of MCPs used in this study (Cunningham TJ et al., 2017), and the method of quantifying total DAPI stained cells and expression of differentiation markers, nearly all of the cells counted were found to

express differentiation markers and therefore are not stuck in a progenitor state. Images in ***Figure 5*** *show confluency in the labeling of cells. We therefore conclude that there is only a change in the ratio of differentiated cell types and not a disruption of differentiation all together.*

4b.) siRNA against HOXC12 and HOXD12 can reverse the effect of siRNA NACA in the iPSC model. What is the specificity of this effect? Quantification of HOXC12 and HOXD12 in treated iPSCs should be evaluated too.

Quantification of HOXC12 and HOXD12 alone is presented in ***Figure 5****. We were not able to test the effect of other HOX genes and include in the discussion the possibility that Nac*α *may also interact with other HOX genes in the iPSC model* *<u>(see lines 391-92).</u>*

<u>Reviewer #2:</u>

1.) The authors next investigate, by treating human iPSCs with siRNAs, whether the same regulatory interaction between Nac-alpha and Hox genes is involved in cardiomyocyte differentiation. There are two apparent Abd-B orthologues in humans, HOXC12 and HOXD12. In these cells, knockdown of Nac-alpha reduces the proportion of cardiomyocytes and increases that of fibroblasts. Knockdown of either HOXC12 or HOXD12 on their own had little effect. In combination with Nac-alpha knockdown, knockdown of either HOXC12, HOXD12, or both, partially rescued the effects of Nac-alpha knockdown to roughly equivalent extents. For me, this result is weak because the double knockdown of HOXC12 and HOXD12 is not examined on its own. It is possible that these genes have redundant functions and the double knockdown would affect the proportions of the various cell types.

*The effect of HOXC12 or HOXD12 single knockdown on cell differentiation are similar. Comparing the combined knockdown between NACA+HOXC12 or NACA+HOXD12 or NACA+HOXC12+HOXD12 also leads to ratios that are similar, therefore we do not believe that there is synergism in the effect of combined HOXC12 and HOXD12. If the reviewer finds it necessary, we can remove the data that includes the combination of NACA, HOXC12 and HOXD12 together (****Figure 5****).*

2.) Also, to further examine whether the regulatory pathway in flies is conserved in humans, the effects of overexpression of HOXC12/HOXD12 should be examined.

While overexpression studies in iPSCs cells would aid in understanding the pathways and interaction between Hox genes and NACA, creating overexpression cell lines are labor and time intensive and would take many months. We have examined in flies the effect of abd-A overexpression and its interaction with NACA, which did not produce a rescue but exacerbated the phenotype and led to lethality during pupal stages (data not shown). We discuss that overexpression of HOXC12 and HOXD12 would be expected to change the ratio of cardiomyocytes and fibroblasts (<u>see lines 393-394</u>).

3.) The final figure examines the effects of disrupting SRP components on cardiac

development. This is of interest because SRP, like NAC, is also recruited to nascent polypeptides. The authors demonstrate that knockdown of various SRP subunits produces a variety of phenotypes, but these results are not extended to the level of any mechanistic insights.

We agree, and in the future, we aim to better understand the role of SRP in heart development. However, in our opinion a detailed examination of SRP mechanism would be beyond the scope of this study, considering the multitude of subunits and exploration of possible targets. (See lines 455-59).

4.) Fig 1D-F: I find it very hard to see the described larval heart phenotypes in these panels. The data are more convincing in Fig S1A. The authors should consider improving the imaging in Fig 1 or substituting the data in Fig S1 to demonstrate the existence of larval hearts in all genotypes.

This figure has been revised. We switched Supplemental Figure 1 live imaging still pictures, which show a larval heart in all three genotypes, and moved it into Figure 1D-F.

5.) Fig S2: The nature of the control should be provided.

We have included details on the genotype of the controls (GD control) in Supplemental Figure 2 (now Supp. Figure 3).

6.) Discussion: This is very long and poorly organized, moving from flies to humans to mice, then back to flies, etc. I think it could be reduced by 50% without substantial loss. The paragraph starting with line 476 includes a fair bit of reiteration of results, the paragraph starting with line 492 could be deleted, and the two paragraphs that discuss Figure 6 are speculative and go far beyond the supporting experimental data. Finally, the presentation of the role of the Nac complex in embryonic patterning in the introduction and discussion contains inaccuracies. Nac is implicated in RNA localization which in turn is required for oskar and nanos translation, so it is not necessarily the case that its effects are only at the level of translation. Relevant to lines 465-468, Oskar protein begins to accumulate at the posterior pole already during mid-oogenesis, not just in the syncytial embryo as this discussion implies.

We have revised and shortened the discussion substantially. We have also revised descriptions of the NAC complex on embryonic patterning in the discussion (see lines 518-24).

Reviewer #3:

1.) RNAi specificity and genotypes. Importantly, the authors use two independent RNAi lines to knock-down Nac-alpha and bic, at least according to the methods. However, Figures 1 and 2 show only the phenotype of one of each without specifying which one was used. Please add the exact genotypes in the figure legend and provide the second RNAi line phenotype in the same figure or in the supplemental figure (at least for Figure 1).

*The RNAi lines have been specified in **Figure 1 and 2.** Examples of additional Nacα and bic RNAi line are also included (**Supplemental Figure 2**), (<u>see lines 179-82</u>). Details of RNAi lines can also be found in **Supplemental Table 1**.*

2. The fact that mis-expression of AbdB in developing pupal hearts is induced as a consequence of NAC knock-down is really interesting. However, it is hard to see on the current image in Figure 3E. Please provide separate grey channel images for the AbdB and the DAPI stains in control and RNAi that we can appreciate the signal in the nuclei.

*We have added the grey channels for ABD-B and DAPI in **Figure 3**.*

3. The authors put forward the interesting idea that NAC is needed specifically in the embryo to prime the heart for later remodelling. This argument largely rests on the finding that knock-down of Nac-alpha during pupal stages only does not result in a strong phenotype. However, it is a well-known phenomenon that knock-down by RNAi depends on the driver used and takes time to be complete. Hence, only knocking-down at pupal stages might simple not reduce the proteins levels of Nac-alpha enough to produce a phenotype. This is consistent with the finding of the authors that the strongest phenotype is found when Nac-alpha is continuously knocked-down or knocked-down in the embryo and in pupae. Thus, the authors may want to explore additional routes to back up their conclusion on this important point. Otherwise, the strength of the phenotype may simply correlate with proteins levels during the remodelling stage.

Possibilities would be to use an antibody against Nac-alpha to show its absence in remodelling pupal hearts following knock-down in pupa only, in contrast to its potential presence in pupae when knocked-down the first 24h or 48h of development only. Alternatives could be using a protein degradation system such us deGradeFP.

We are able to see cardiac defects in adult hearts following KD of Nacα for just 24 hours in the embryo, suggesting 24 hrs in the embryo was sufficient to cause cardiac defects in the adult. The number of days of knockdown needed for Embryo + pupae condition and loss of the heart is about the same length of knockdown used for mid-larvae through pupae until eclosion, which did not show any heart loss (Fig. 4E). Furthermore, the KD from mid-larvae through pupae until eclosion is a continuous knockdown, unlike Embryo+pupae. One should expect that the continuous knockdown would be more effective than discontinuous KD to affect cardiac remodeling. Despite this, mid-larvae to eclosion still did not elicit heart loss. We have included the number of days of knockdown for clarity (<u>See lines 315-326</u>).

*To address this further, we have performed cardiac qPCR to examine Nacα mRNA levels in the hearts of early pupae following embryo only knockdown, to see if embryo knockdown only has altered Nacα levels in early pupae, that may contribute to the heart loss when Nacα is knocked also down in pupae stages. Indeed, the reduced levels of Nacα were observed, despite the lack of Nacα-RNAi induction at that stage (new **Supplemental Figure 8**, right bars). This suggests that embryonic KD only leads to long-term cardiac remodeling, including reduced Nacα RNA levels at the early pupal stage. However, without*

*further reduction of Nac α RNA during pupal stages (new **Supplemental Figure 8**, left bars), this only elicits partial heart loss (Fig. 4E vs 4H). Only with further Nac α-RNAi induction can a near complete heart loss be induced. (See lines 332-41) As mentioned above, we have also found reduced Nac α mRNA levels in young adults following Nac α KD during pupae stages only (new **Supp Fig 8**). This would suggest that Nac α KD during pupation, as measured in early adults, does not by itself lead to loss of the heart (Fig. 4D), as does the combination of early and pupal knockdown (see lines 329-331).*

Of note, the Nac α RNA levels measured by qPCR are likely an underestimate, since the heart dissections include modest amounts of cells where the Hand4.2 driver is not expressed, including fat, hemocytes and muscle cells (alary muscles).

There is unfortunately no available Nac α antibody that works convincingly in Drosophila. We obtained an aliquot designed for the human homolog, but we were not able to detect reliable staining using the antibody.

4. Figure 5 is missing a control for effectiveness of NACA RNAi when combined with a second siRNA against an unrelated gene. Any of the combinations shown at the moment appear to rescue, even though this rescue is not very obvious in the overview images. Would higher magnifications help?

*We have improved the images in new **Figure 5** and used higher magnification of the images to show the shifts in cell populations.*

*We have in previous studies (**Schroeder et al 2019**) used combinatorial siRNA-mediated KD approaches in MCPs to check differentiation phenotypes, and the level of knockdown of individual genes is not much altered even in the presence of additional siRNA combinations. (See lines 387-89)*

5. Can the different phenotypes seen after knock-down of the various SRP subunits not simply be explained by varying effectiveness of knock-down? Again, no second RNAi lines are shown and no protein amounts were measured, hence the shown phenotypes are very hard to interpret. In my opinion, it would take a significant amount of more work to support the hypothesis of the authors that the different SRP subunits do different things in the different heart cells. Properly investigating this hypothesis will take an entire manuscript on its own. How is the phenotype when the same RNAi lines were used in skeletal muscle cells?

We agree that some of the phenotypes could be due to expression level and that a detailed study of individual SRP subunits would be beyond the scope of this study.

*We have revised the results and discussion to tone down our interpretation of the role of SRPs on the heart (see lines 455-59). We have included a list of all the RNAis used for each SRP gene (See **Supplemental Table 1**).*

6. The discussion is overly long.

We have much shortened the discussion.

Minor points.
1. The heart remodelling movies are really nice. Please include a time stamp in the movies. Compression of the 70MB large movie files would be useful.

We have included timestamps and compressed the movies.

2. Figure 2C and F, please shift the driver labels a bit to the left or change the organisation of the labels. Hard to grasp which RNAi was combined with which driver in which of the graphs. Please label micro with µ. What is a KK control? An unrelated RNAi line? Were no GD lines used?

*We have adjusted the labels on **Figure 2C-2H.** KK control is the background fly line wherein the RNAi construct was inserted, and therefore used as an appropriate background control. We have clarified this in the methods (lines 586-7). We also included examples of cardiac phenotypes using additional RNAi lines (GD) for Nacα and Bic (new **Supplemental Figure 2**; lines 179-182)*
